# Evaluation of etoricoxib in patients undergoing total knee replacement surgery in a double-blind, randomized controlled trial

**DOI:** 10.1186/1471-2474-14-300

**Published:** 2013-10-24

**Authors:** Narinder Rawal, Eugene Viscusi, Paul M Peloso, Harold S Minkowitz, Liang Chen, Sandhya Shah, Anish Mehta, Denesh K Chitkara, Sean P Curtis, Dimitris A Papanicolaou

**Affiliations:** 1 Department of Anaesthesiology and Intensive Care, Orebro University Hospital, Örebro, SE 701 85, Sweden; 2 Department of Anesthesiology, Thomas Jefferson University, Philadelphia, PA, USA; 3 Clinical Research, Merck & Co., Inc, Whitehouse Station, NJ, USA; 4 Memorial Hermann Memorial City Medical Center, Houston, TX, USA; 5 Late Development Statistics, Merck & Co., Inc, Whitehouse Station, NJ, USA; 6 Office of the Chief Medical Officer, Merck & Co., Inc, Whitehouse Station, NJ, USA

**Keywords:** Etoricoxib, Ibuprofen, Morphine consumption, Total knee replacement, Pain at rest, Pain upon movement

## Abstract

**Background:**

Optimal postoperative pain management is important to ensure patient comfort and early mobilization.

**Methods:**

In this double-blind, placebo- and active-controlled, randomized clinical trial, we evaluated postoperative pain following knee replacement in patients receiving placebo, etoricoxib (90 or 120 mg), or ibuprofen 1800 mg daily for 7 days. Patients ≥18 years of age who had pain at rest ≥5 (0–10 Numerical Rating Scale [NRS]) after unilateral total knee replacement were randomly assigned to placebo (N = 98), etoricoxib 90 mg (N = 224), etoricoxib 120 mg (N = 230), or ibuprofen 1800 mg (N = 224) postoperatively. Co-primary endpoints included Average Pain Intensity Difference at Rest over Days 1–3 (0- to 10-point NRS) and Average Total Daily Dose of Morphine over Days 1–3. Pain upon movement was evaluated using Average Pain Intensity Difference upon Knee Flexion (0- to 10-point NRS). The primary objective was to demonstrate analgesic superiority for the etoricoxib doses vs. placebo; the secondary objective was to demonstrate that the analgesic effect of the etoricoxib doses was non-inferior to ibuprofen. Adverse experiences (AEs) including opioid-related AEs were evaluated.

**Results:**

The least squares (LS) mean (95% CI) differences from placebo for Pain Intensity Difference at Rest over Days 1–3 were -0.54 (-0.95, -0.14); -0.49 (-0.89, -0.08); and -0.45 (-0.85, -0.04) for etoricoxib 90 mg, etoricoxib 120 mg, and ibuprofen, respectively (p < 0.05 for etoricoxib vs. placebo). Differences in LS Geometric Mean Ratio morphine use over Days 1–3 from placebo were 0.66 (0.54, 0.82); 0.69 (0.56, 0.85); and 0.66 (0.53, 0.81) for etoricoxib 90 mg, etoricoxib 120 mg, and ibuprofen, respectively (p < 0.001 for etoricoxib vs. placebo). Differences in LS Mean Pain Intensity upon Knee Flexion were -0.37 (-0.85, 0.11); -0.46 (-0.94, 0.01); and -0.42 (-0.90, 0.06) for etoricoxib 90 mg, etoricoxib 120 mg, and ibuprofen, respectively. Opioid-related AEs occurred in 41.8%, 34.7%, 36.5%, and 36.3% of patients on placebo, etoricoxib 90 mg, etoricoxib 120 mg, and ibuprofen, respectively.

**Conclusions:**

Postoperative use of etoricoxib 90 and 120 mg in patients undergoing total knee replacement is both superior to placebo and non-inferior to ibuprofen in reducing pain at rest and also reduces opioid (morphine) consumption.

**Clinical trial registration:**

NCT00820027

## Background

Total knee replacement surgery is an option taken by patients who suffer from debilitating pain and loss of function, in association with demonstrated loss of cartilage due to a variety of conditions, but most commonly associated with osteoarthritis. The procedure has established efficacy and provides pain relief as well as functional improvement [1-3]. Additionally, the frequency of this procedure is increasing in the Western world as the population ages and the prevalence of osteoarthritis rises [4,5].

Postoperative pain management serves several goals including reduction of suffering and improvement of recovery. After the initial immediate postoperative period, good pain management also improves quality of life and satisfaction with the procedure [6]. Multimodal pain management strategies that maximize pain relief while minimizing tolerability issues such as opioid-related adverse events (AEs) are recommended postoperatively through the modulation of several pain pathways at lower doses than would be required if a single pathway were targeted [7-10]. Although non-steroidal anti-inflammatory drugs (NSAIDs) provide good complementary effects for pain relief when combined with opioids in multimodal analgesia regimens, guidelines and common practice generally recommend patients discontinue them. These recommendations are due to the inhibition of cyclo-oxygenase (COX)-1 by traditional NSAIDs, which has been shown to interfere with platelet aggregation and can thus lead to the possibility of increased bleeding in surgical settings.

The present study evaluates the use of etoricoxib, a selective COX-2 inhibitor, as part of a multimodal analgesic treatment in total knee replacement surgery patients. Etoricoxib has demonstrated efficacy in many painful conditions, including a previous study that demonstrated efficacy in patients undergoing total knee or hip arthroplasty [11]. Its pharmacokinetic profile shows a lack of effect on COX-1, and, therefore, it has minimal effects on platelet aggregation [12] making it an appropriate treatment in surgical settings in contrast to traditional NSAIDs such as ibuprofen.

This study provides additional information on the efficacy and safety of etoricoxib in a large patient sample treated for 7 days postoperatively and followed for 21 days after the first dose; two doses of etoricoxib (90 and 120 mg) were evaluated against both an active comparator (ibuprofen) and placebo, as well as assessing both static pain and pain on movement. The hypothesis of this trial was that etoricoxib 90 and 120 mg would provide superior efficacy compared with placebo and would be non-inferior to ibuprofen.

## Methods

This study (Sponsor Protocol #098) was registered at clinicaltrials.gov (NCT00820027). It was conducted at 63 sites globally, including in the United States (13 sites); Europe (41 sites, includes Bulgaria, Czech Republic, Estonia, Germany, Hungary, Lithuania, Norway, Serbia, Slovenia, South Africa and Turkey); Costa Rica (1 site); and Asia Pacific (8 sites, includes Philippines, South Korea, Singapore and Taiwan) from December 30, 2008 to December 13, 2010. All patients gave written informed consent prior to initiation of the study, and the protocol and all amendments were approved by local institutional review boards (IRBs). The specific institutional review boards that approved the study are provided in (Additional file 1). The study was conducted in accordance with the principles of Good Clinical Practice and applicable country and/or local statutes and regulations.

### Patients

Eligible patients were men and women, at least 18 years of age, considered to be in good general health, and scheduled to have a unilateral total knee replacement completed in 3 hours or less. Chronic, stable health conditions were permitted. Women of childbearing potential were required to have a β-hCG consistent with a non-gravid state prior to randomization and were then required to use adequate birth control methods or remain abstinent during the study. Post-operatively, patients needed to tolerate clear liquids and have a pain intensity of ≥5 (0- to 10-point numerical rating scale [NRS] where 0 = no pain and 10 = pain as bad as you can imagine) in the recovery room.

Patients were excluded if they were allergic or intolerant to study medications; had a recent history of chronic drug abuse/dependence; had received an investigational agent/device (within 4 weeks); or were morbidly obese. Patients with certain cardiovascular (uncontrolled hypertension, congestive heart failure, history of coronary artery bypass graft surgery, angioplasty, cerebrovascular accident or transient ischemic attack) and gastrointestinal conditions (gastric ulcer, gastric surgery, or inflammatory bowel disease) were also excluded.

### Study design

This was a randomized, double-blind, placebo- and active-comparator-controlled, multiple-dose study performed under in-house blinding in patients undergoing unilateral total knee replacement surgery to evaluate the tolerability and efficacy of etoricoxib. The first dose of study medicine (oral etoricoxib 120 mg, etoricoxib 90 mg, ibuprofen dose of 600 mg or placebo) was administered post-operatively in the recovery room and prior to 6 p.m. on the day of surgery. To maintain the blinding, all therapies were given 3 times a day (with 1 active dose of etoricoxib and 2 doses of placebo), to mimic the total daily dosing frequency of ibuprofen.

At the time of the first post-operative dose of study medication, patients were connected to a patient-controlled analgesia (PCA) device and morphine was self-administered for at least 24 hours, as needed to control pain. The PCA device was set to deliver 1 mg morphine with a 6-minute lock-out period and a maximum total dose of 40 mg in 4 hours. If the dose was inadequate, the dose could have been titrated incrementally up to 2 mg for a maximum total dose of 50 mg in 4 hours. Patients were disconnected from the PCA device when it was no longer needed; defined as a time lapse since the last PCA morphine dose ≥2 hours and a patient’s pain intensity reported as ≤4 (0-to-10-point NRS). After disconnecting the PCA device, patients could supplement their analgesia with 2 oxycodone 5 mg tablets up to 4 times a day (daily maximum of 80 mg oxycodone), through Day 7, in addition to their study medication.

On Days 2 and 3, the first dose of study medication was given approximately 24 and 48 hours after the initial dose taken on Day 1, respectively. Patients received their second and third doses of ibuprofen 600 mg or matching placebo every 8 hours from the time of the first dose. On Days 4 to 7, patients were switched to a first dose of study medication at 8 a.m, with second and third doses of ibuprofen 600 mg or matching placebo given every 6 hours, (i.e. 2 p.m. and 8 p.m.). There were 10 study visits: Visit 1 (screening visit within 21 days of surgery date); Visit 2 (postsurgery predose); Visit 3 (randomization); Visit 4 was for database purposes only and was not a separate clinic/hospital visit; Visits 5–9 (days 3,4,5,6,7); Visit 10 (one day after last dose of study medication).

### Surgical procedure

Knee replacement surgery was performed according to local practice, but anesthesia was standardized using either general or spinal anesthesia. General anesthesia consisted of isoflurane, sevoflurane, or desflurane and nitrous oxide. Spinal anesthesia consisted of up to 15 mg bupivacaine with no spinal opioids. All patients received 4 mg of ondansetron IV during induction to prevent postoperative nausea and vomiting. Propofol was allowed at a dose of 2 to 3 mg/kg and fentanyl was not to exceed a cumulative intra-operative dose of 10 ug/kg or a total cumulative dose of 500 ug, whichever was less. Infiltration of the wound with any local anesthetic agent, including intra-articular analgesia, was not permitted.

### Study medication dosing

Patients were allocated at random based on a computer-generated randomization schedule by an Interactive Voice Response System (IVRS) on the day of admission for surgery, to 1 of the 4 treatment options. The active medications and placebo were identical in appearance, size, shape, and taste, ensuring proper blinding of patients, investigator study staff, and sponsor staff, throughout the study duration. Patients were required to stay in hospital for at least 72 hours from the first dose of study medication.

### Efficacy measurements and hypotheses

The co-primary endpoints were Average Pain Intensity Difference at Rest (as measured on a 0- to 10-point NRS) over Days 1 to 3 and Average Total Daily Dose of Morphine over Days 1 to 3. Other endpoints included Change from Baseline in Average Pain Intensity at Rest (0- to 10-point NRS) over Days 4 to 7, Average Total Daily Dose of Morphine Consumed over Days 4 to 7, and Change from Baseline in Average elicited Pain Intensity at Knee Flexion over Days 1 to 3 and over Days 4 to 7.

The objectives of this study were to 1) demonstrate that the analgesic effect of etoricoxib 90 mg or 120 mg, administered once daily, is superior to placebo for the treatment of pain following total knee replacement orthopedic surgery (primary objective); 2) to demonstrate that the analgesic effect of etoricoxib 90 mg or 120 mg, administered once daily, is non-inferior to ibuprofen 1800 mg; and 3) to assess the safety and tolerability of repeated doses of etoricoxib administered over a total 7-day time period in patients treated for pain following total knee replacement orthopedic surgery.

To address these objectives, the primary hypotheses were that: 1) the Average Pain Intensity Difference at Rest over Days 1 through 3 in patients treated with etoricoxib 90 mg or 120 mg would be superior to placebo; 2) the Average Total Daily Dose of Morphine on Days 1 through 3 in patients treated with etoricoxib 90 mg or 120 mg would be less than in patients treated with placebo; and 3) etoricoxib 90 mg and 120 mg would be generally safe and well tolerated. The secondary hypothesis was that the Average Pain Intensity Difference at Rest over Days 1 through 3 in patients treated with etoricoxib 90 mg or 120 mg would be non-inferior to that of ibuprofen 1800 mg. Ibuprofen was included in the study as an active comparator; therefore, there was no planned statistical comparison for ibuprofen compared with placebo.

### Pain intensity at rest

Patients rated their pain at rest while in a supine position. During the treatment period, patients recorded Pain Intensity at Rest at 4 pre-specified time points on Days 1 to 3 and at 2 pre-specified time points on Days 4 to 7.

### Pain intensity at knee flexion

Patients rated Pain Intensity with Knee Flexion, on a 0- to 10-point NRS; a baseline measurement on the knee that underwent joint replacement was taken prior to administering the first dose of study medication. The Pain Intensity with Knee Flexion assessment was done 2 hours after the first dose of study medication on Days 1 to 3 and once between 12 to 4 pm on Days 4 to 7. Knee flexion was performed passively, to 90 degrees.

### Opioid use

The Average Total Daily Dose of Morphine (morphine equivalent) used during Days 1 to 3 was a co-primary endpoint and the average Total Daily Dose of Morphine (morphine equivalent) used during Days 4 to 7 was a tertiary endpoint for the study. The total included all doses of morphine (delivered by PCA and IV bolus), oxycodone 5 mg, and any other opioids.

The Recovery Index (RI), a 49-item questionnaire, was included to monitor patient recovery postoperatively. The Opioid Side Effects Scale is a 22-item subscale of the RI-49 that was used to assess patient tolerance of opioid side effects. Patients completed the RI Scale on Days 2 to 7 at bedtime. Patients also completed the RI Scale at the discontinuation visit if they had not completed the RI Scale in the past 12 hours.

Other efficacy endpoints included Average Pain Intensity Difference at Rest (0- to 10-point NRS) over Days 4 to 7, Average Total Daily Dose of Morphine (morphine equivalent) over Days 4 to 7, Average Elicited Pain Intensity Difference at Knee Flexion (0- to 10-point NRS) over Days 1 to 3 and Average Elicited Pain Intensity Difference at Knee Flexion (0- to 10-point NRS) over Days 4 to 7.

### Responder analyses

*Post hoc* responder analyses were conducted to provide further insight on the effect of etoricoxib compared with placebo by evaluating the level of improvement achieved from Days 1 to 3 and from Days 4 to 7, consistent with the Initiative on Methods, Measurement, and Pain Assessment in Clinical Trials (IMMPACT) recommendations for reporting on clinical trials [13]. Percent change from baseline over Days 1 to 3 and percent change from baseline over Days 4 to 7 were calculated based on the Pain Intensity at Rest (0- to-10 point NRS) and the Pain Intensity at Knee Flexion (0- to-10 point NRS). The percentages of patients reaching various degrees of improvement (i.e., percent reductions) in the Pain Intensity at Rest (0-to-10 point NRS) and in the Pain Intensity at Knee Flexion (0-to-10 point NRS) were plotted by treatment groups. Because Pain Intensity at Rest was measured on a 0- to 10-point NRS scale, a negative change from baseline indicated an improvement from baseline. Patients who dropped out at any day of Days 1 to 3 or of Days 4 to 7 for any reason were assigned an improvement of 0% for the corresponding responder analysis.

### Subgroup analyses

In order to explore the consistency of the treatment effects with respect to baseline pain intensity (moderate versus severe pain) and type of anesthesia (spinal versus general) patients received for the surgery, subgroup analyses were performed for the 2 co-primary endpoints.

### Safety measurements

Safety and tolerability were assessed through physical examination, vital signs, laboratory tests (CBC, serum chemistry, urinalysis, and urine pregnancy tests). In addition, this study was subject to a prespecified blinded, adjudication procedure for potential thromboembolic, investigator-reported serious cardiovascular AEs.

Safety assessments were summarized using a tiered approach [14]. Tier 1 AEs were prespecified and included edema-related AEs; hypertension-related AEs; a composite AE of congestive heart failure, pulmonary edema or cardiac failure; and opioid-related AEs (i.e., nausea, vomiting, constipation, somnolence, respiratory depression, urinary retention, and ileus). Tier 2 AEs included any adverse event not prespecified as Tier 1 that occurred in ≥4 patients in any one of the treatment groups. Tier 3 AEs included change from baseline in vital signs, serum creatinine, and estimated glomerular filtration rate; and any adverse event not prespecified as Tier 1 or Tier 2 that occurred in <4 of patients in all of the treatment groups.

### Statistical analyses

The study plans called for 713 patients to be randomized in a fully concealed manner (i.e. blinded to patient, study staff, sponsor personnel, etc.) to 1 of 4 treatments in a 9:9:9:4 ratio: etoricoxib 120 mg (n = 207), etoricoxib 90 mg (n = 207), ibuprofen 1800 mg (600 mg TID) (n = 207) or placebo (n = 92), with a total of 776 ultimately randomized. For the co-primary endpoint of Average Pain Intensity at Rest over Days 1 to 3, this sample size was predicted to have > 99% power (α = 0.05, 2-tailed) to detect a significant difference between etoricoxib and placebo of 2 points, with an assumed standard deviation of 3.08. For the second co-primary endpoint, Average Total Daily Dose of Morphine over Days 1 to 3, the study was powered at 93% to detect a 25% reduction in morphine use (α = 0.05, 2-tailed) between etoricoxib and placebo doses.

For the non-inferiority comparison of etoricoxib (120 mg, 90 mg) to ibuprofen 1800 mg with respect to the average pain intensity difference over Days 1 to 3, with an assumed standard deviation of 3.08, the sample sizes were predicted to have 91% power (1-sided, α =0.025) to declare that etoricoxib was at least as effective as ibuprofen 1800 mg, given a non-inferiority bound of 1 point on a 0–10 NRS.

To preserve the experimental-wise type-I error rate for the co-primary and secondary hypotheses, the comparisons of the 2 etoricoxib doses with placebo were conducted in a step-down manner. Etoricoxib 120 mg was compared with placebo with the co-primary endpoints first, and, only if it was shown to be superior to placebo at the 5% critical level (2-sided) for each of the 2 co-primary endpoints was the 90-mg dose compared with placebo, also at the 5% critical level for each of the co-primary endpoints (2-sided). Because both co-primary endpoints had to be significant (α = 0.05, two-sided) in order to declare that an etoricoxib dose (120 mg, 90 mg) was superior to placebo, no multiplicity adjustment was needed for the co-primary endpoints for the given dose. This step-down testing procedure controls the type-I error rate at 5% for the testing of both doses against placebo for the co-primary hypotheses. The secondary hypothesis of the non-inferiority of an etoricoxib dose to ibuprofen 1800 mg was conducted with regard to the Average Pain Intensity Difference at Rest (0- to 10-point NRS) over Days 1 to 3 only if that same etoricoxib dose was shown to be superior to placebo with respect to both of the co-primary endpoints at the critical 5% level.

The Full Analysis Set (FAS) population, including all randomized patients who received at least 1 dose of study medication and had at least 1 post-randomization measurement, was used for the primary efficacy analysis. The Per-Protocol (PP) population was used as a supportive analysis.

A longitudinal data analysis (LDA) [15] was used to analyze between-group differences in Average Pain Intensity difference at Rest (0- to 10-point NRS) over Days 1 to 3 and over Days 4 to 7 and Average Elicited Pain Intensity difference at knee flexion (0- to 10-point NRS) over Days 1 to 3 and over Days 4 to 7. This LDA model included both baseline and post-baseline measurements as response variables. The LDA model included terms for treatment, time, and the interaction of time by treatment, with restriction of the same baseline mean across treatment groups (due to randomization). The LDA model also adjusted for baseline pain intensity and anesthesia type used for surgery. A longitudinal ANOVA was used to analyze between-group differences in the Average Total Daily Dose of Morphine over Days 1 to 3 and morphine (morphine equivalent) over Days 4 to 7 and the Recovery Index Opioid Side Effects Score on each day from Day 2 to 7. The longitudinal ANOVA model included terms for baseline pain intensity, anesthesia types, treatment, time, and the interaction of time by treatment. Log-transformation was applied to the total daily morphine use to bring them to a relative scale. The average total daily dose of morphine over Days 1 to 3 and Days 4 to 7 was estimated from the longitudinal ANOVA model and back-transformed as LS geometric means.

A Cox proportional hazards regression model was used to analyze time to last dose of morphine administered via PCA device; between-group comparisons were assessed by risk ratios (with 95% confidence intervals [CIs]). Time-to-event endpoints were plotted using Kaplan-Meier curves.

The All Patients as Treated (APaT) population was used for safety analysis and included all randomized patients who received ≥1 dose of study drug and had a change from baseline in laboratory measurements.

For the responder analyses, the differences between each dose of etoricoxib and the comparators and between the two doses regarding the percentages for patients achieving a ≥20%, ≥50%, and ≥70% improvement from baseline based on the Pain Intensity at Rest over Days 1 to 3 were tested separately using the Miettinen and Nurminen method, an unconditional, asymptotic method with α = 0.050 (2-sided) [16]. For the subgroup analyses, the evaluation was primarily through the examination of the LS means for the treatment groups across the different categories or levels of the pre-existing factor and the associated nominal 95% confidence intervals.

### Safety analyses

The analysis of safety results followed a tiered approach; the tiers differed with respect to the analyses performed. Safety parameters or AEs of special interest that were identified *a priori* were considered Tier 1 safety endpoints and were subject to inferential testing for statistical significance. P-values (Tier 1 only) and 95% CIs (Tier 1 and Tier 2) were provided for between-treatment differences in the percentage of patients with events; these analyses were performed using the Miettinen and Nurminen method [16].

## Results

### Patients

**Figure 1:**
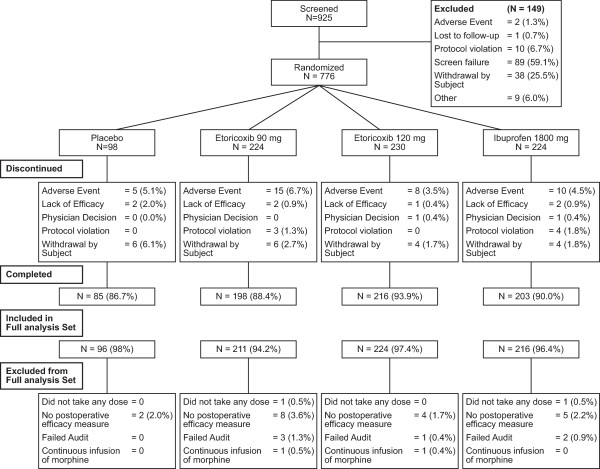
CONSORT Diagram showing patient accounting.

Of the 925 patients screened for the study, 776 patients met the study inclusion criteria and were randomized to placebo (N = 98), etoricoxib 90 mg (N = 224), etoricoxib 120 mg (N = 230), or ibuprofen (N = 224) (Figure 1). The primary reasons for exclusion from the trial included failure to meet inclusion criteria and patient withdrawal. There was one study site that failed audit, and therefore the 6 patients from that site were excluded from final analyses. In total, 702 (90.5%) patients completed the study. The rates of completion and discontinuation were similar for all treatment groups. The primary reasons for study discontinuation were related to adverse experiences and patient withdrawal (Figure 1).

All treatment groups were similar with respect to baseline demographics (Table 1). Overall, patients underwent surgery lasting a mean of 1.4 hours and had a mean baseline pain intensity score at rest (0- to 10-point NRS) of 7.0 (after surgery and before randomization to study medication). A majority of patients (62.2%) underwent surgery after spinal anesthesia.

**Table 1:** Baseline characteristics

	**Placebo**	**Etoricoxib 90 mg**	**Etoricoxib 120 mg**	**Ibuprofen 1800 mg**
	**N = 98**	**N = 224**	**N = 230**	**N = 224**
Female n (%)	56 (57.1)	134 (59.8)	139 (60.4)	152 (67.9)
Age				
Mean [yr (SD)]	65.2 (7.9)	65.7 (8.5)	64.7 (8.1)	66.0 (8.1)
Median	64.5	66.0	64.0	66.0
Range	43 - 81	37 - 84	44 - 86	38 – 84
Height in cm (SD)	167.1 (10.6)	166.9 (10.8)	168.1 (10.6)	165.8 (10.6)
Body Weight in kg (SD)	85.9 (17.3)	86.2 (17.6)	85.4 (16.2)	84.4 (16.6)
Body Mass Index (SD)	30.6 (4.7)	30.8 (5.0)	30.2 (4.7)	30.6 (4.7)
Duration of Surgery in hours (SD)	1.4 (0.5)	1.4 (0.5)	1.4 (0.5)	1.4 (0.5)
Baseline Pain Intensity at Rest NRS (SD)				
Mean Score (SD)	7.1 (1.8)	7.0 (1.8)	7.1 (1.8)	6.9 (1.7)
Mild Pain (Score: 0–4) n (%)	0 (0.0)	1 (0.4)	0 (0.0)	1 (0.4)
Moderate Pain (Score 5–7) n (%)	60 (61.2)	137 (61.2)	135 (58.7)	140 (62.2)
Severe Pain (Score 8–10) n (%)	38 (38.8)	85 (37.9)	94 (40.9)	83 (37.1)
Anesthesia Type n (%)				
General	38 (38.8)	83 (37.1)	90 (39.1)	81 (36.2)
Spinal	60 (61.2)	141 (62.9)	140 (60.9)	142 (63.4)
Other	0 (0.0)	0 (0.0)	0 (0.0)	1 (0.4)

### Efficacy endpoints

#### Average pain intensity at rest

**Figure 2:**
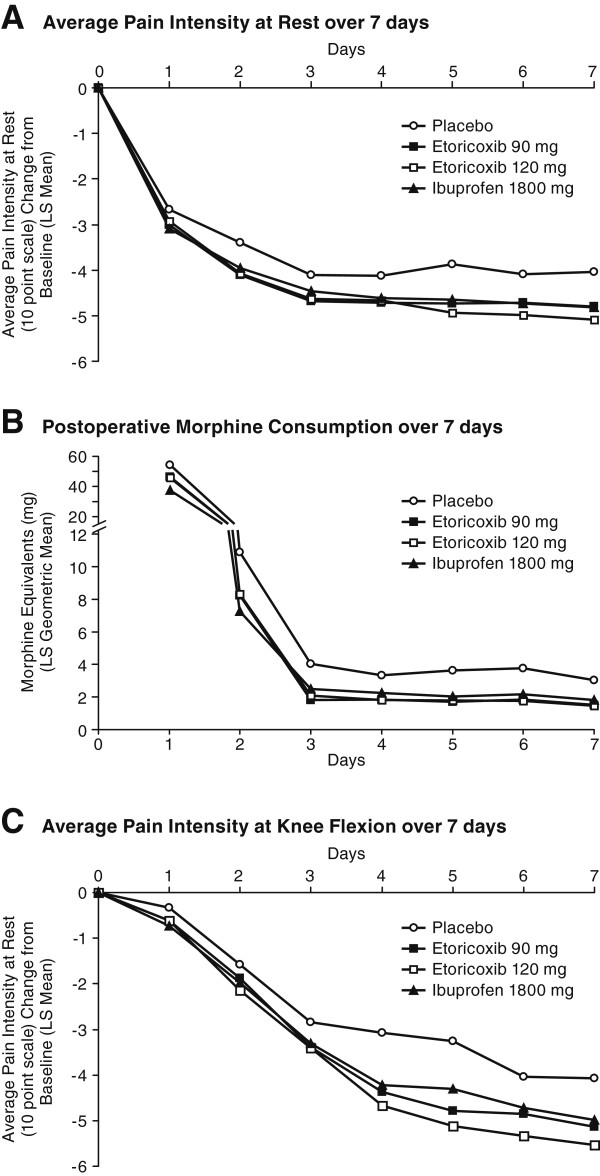
**Co-primary efficacy endpoints over 7 Days.** Panel **A** shows Average Pain Intensity at Rest over 7 days. Panel **B** shows Postoperative Morphine Consumption over 7 days. Panel **C** shows Average Pain Intensity at Knee Flexion over 7 days.

Both etoricoxib doses (120 mg and 90 mg) were superior to placebo (p = 0.018 and p = 0.009, respectively) and also non-inferior to ibuprofen 1800 mg daily. In the co-primary endpoint of change from baseline in Average Pain Intensity at Rest over Days 1 to 3, the improvements seen for the 2 etoricoxib dose groups (120- and 90-mg) were similar (Table 2; Figure 2A).

**Table 2:** Summary of efficacy endpoints

**Endpoints**	**Placebo**	**Etoricoxib 90 mg**	**p-Value vs. placebo**	**Etoricoxib 120 mg**	**p-Value vs. placebo**	Ibuprofen 1800 mg*
***Primary endpoints***	***Value (95% CI)***					
LS Mean Change from Baseline Average Pain Intensity at Rest over Days 1–3 (0–10 point NRS)	-3.39 (-3.74, -3.04)	-3.93 (-4.17, -3.69)	*p = 0.018*	-3.87 (-4.11, -3.64)	*p = 0.009*	-3.83 (-4.07, -3.59)
LS Geometric Mean Total Daily Dose of Morphine Over Days 1 to 3 mg	13.4 (11.2, 16.0)	8.87 (7.88, 9.97)	*p < 0.001*	9.25 (8.26, 10.4)	*p < 0.001*	8.82 (7.85, 9.91)
Raw Geometric Mean Total Daily Dose Over Days 1 to 3	13.5	8.84	*N/A*	9.26	*N/A*	8.83
***Other endpoints***						
LS Mean Change from Baseline Average Pain Intensity at Rest over Days 4–7 (0–10 point NRS)	-4.03 (-4.39, -3.67)	-4.74 (-5.00, -4.49)	*p < 0.001*	-4.92 (-5.16, -4.67)	*p < 0.001*	-4.70 (-4.95, -4.45)
Postoperative Morphine Consumption (mg)	3.43 (2.69, 4.36)	1.72 (1.46, 2.02)	*N/A*	1.70 (1.46, 1.99)	*N/A*	2.06 (1.76, 2.41)
LS Geometric Mean Total Daily Dose Over Days 4 to 7						
Raw Geometric Mean Total Daily Dose Over Days 4 to 7	3.49	1.71	*N/A*	1.70	*N/A*	2.06
LS Mean Change from Baseline in Pain Intensity at Knee Flexion over Days 1 to 3 (0- to 10-point NRS)	-1.59 (-2.00, -1.17)	-1.96 (-2.24, -1.68)	*p = 0.057*	-2.05 (-2.32, -1.78)	*p = 0.129*	-2.00 (-2.28, -1.72)
LS Mean Change from Baseline in Pain Intensity at Knee Flexion over Days 4 to 7 (0- to 10-point NRS)	-3.61 (-4.05, -3.18)	-4.78 (-5.08, -4.48)	*p < 0.001*	-5.16 (-5.45, -4.87)	*p < 0.001*	-4.56 (-4.85, -4.26)

*p-values were not calculated for the comparison of ibuprofen vs. placebo.

### Morphine utilization

Both etoricoxib doses (120 mg and 90 mg) were superior to placebo in the co-primary endpoint of average total daily dose of morphine over Days 1 to 3 (p < 0.001); morphine consumption was 31% and 34% less per day, averaged over the first 3 days, in patients treated with etoricoxib 120 mg and 90 mg, respectively, vs. placebo (Table 2). No notable differences in average daily morphine consumption over Days 1 to 3 occurred between etoricoxib 120 mg (-4.15 mg vs. placebo) or etoricoxib 90 mg (-4.53 mg vs. placebo) and ibuprofen 1800 mg (-4.58 mg vs. placebo). The morphine consumption amounts among the patients administered etoricoxib 120 and 90 mg were approximately 15% less than placebo on Day 1 and this difference increased to ~25% on Day 2 and between 45% to 55% on Days 3 to 7 (Figure 2B).

### Pain on movement

When averaged over Days 1 to 3, etoricoxib 120 mg and 90 mg showed greater reductions from baseline in Pain Elicited with Knee Flexion, with reductions of 0.46 and 0.37 units on a 0-to-10 NRS scale, respectively, compared with placebo; however, the differences were not statistically significant (nominal p values ≥0.057).

Averaged over Days 4 to 7, both etoricoxib dose groups (120 mg, 90 mg) showed nominally significant greater reduction from baseline as compared with placebo on Pain Intensity with Knee Flexion.

There were no notable differences observed when comparing etoricoxib doses (120 mg, 90 mg) with ibuprofen 1800 mg in average change from baseline in Pain Intensity at Knee Flexion over Days 1 to 3; however, greater differences between etoricoxib 120 mg and ibuprofen were observed over Days 4 to 7 in favor of etoricoxib 120 mg (i.e., exclusion of 0 in the confidence intervals) (Figure 2C).

### Responder analyses

**Figure 3:**
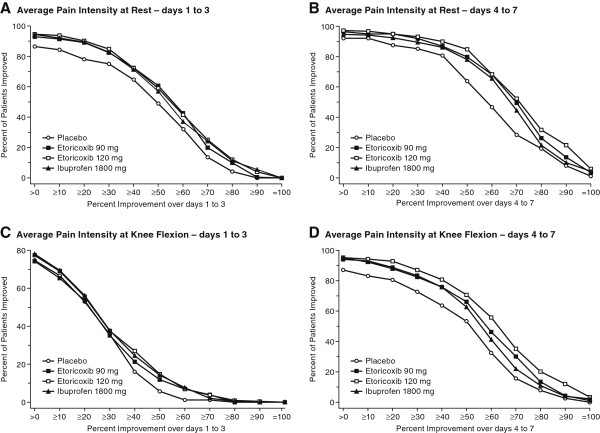
Responder Analyses showing the percent of patients who improved by different percent levels of pain intensity reduction at rest on Days 1 to 3 (Panel A) and Days 4 to 7 (Panel B) and at knee flexion on Days 1 to 3 (Panel C) and Days 4 to 7 (Panel D).

Analyses were performed to optimize assay sensitivity and identify patients who attained levels of pain reduction consistent with being clinically important (i.e., 30%, 50%, and 70% improvement). Figure 3 demonstrates that greater improvements and greater separation from placebo were achieved with study medication for both Average Pain Intensity at Rest (Figures 3A and B) and Average Pain Intensity at Knee Flexion (Figures 3C and D) for the Day 4 to 7 period as compared with the Day 1 to 3 period.

### Subgroup analyses – average pain intensity at rest and morphine consumption by subgroups of patients with different levels of baseline pain severity and type of anesthesia

**Figure 4:**
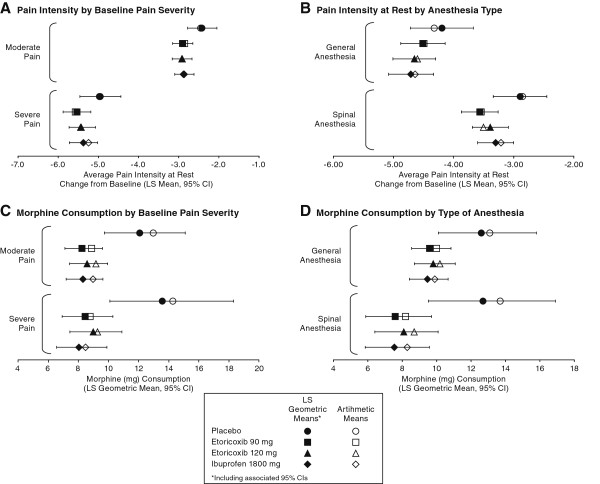
**Subgroup analyses for the co-primary endpoints of patients who had moderate pain vs. severe pain (Panels A and B) and for patients who received general anesthesia vs. spinal anesthesia (Panels C and D).** Both LS Geometric Means (solid squares) and associated 95% CIs as well as Arithmetic Means (open circles) are shown.

Patients who had severe pain at baseline had a greater improvement from baseline than those who began the study with moderate pain at baseline. However, the ranking of improvement among the active treatments (etoricoxib and ibuprofen) and placebo were consistent across these subgroups (Figure 4A).

Patients who received general anesthesia reported higher levels of baseline pain at the time of randomization. Further, the improvement from baseline was also larger for patients receiving general anesthesia as compared with those who had spinal anesthesia; this was true across all treatments and the rank of the treatment differences from placebo for the 3 active treatment groups (etoricoxib and ibuprofen) was also consistent across the anesthesia subgroups (Figure 4B).

Interestingly, there was no important difference in the amount of morphine consumed based on baseline pain severity (Figure 4C). There was numerically less consumption of morphine in patients receiving general anesthesia as compared with spinal anesthesia, for the active therapies, although the placebo groups had similar morphine consumption (Figure 4D).

### Safety and tolerability

The incidence of clinical AEs was generally similar among the placebo, etoricoxib (120 mg, 90 mg), and ibuprofen groups; overall, few serious AEs (2.3%) and drug-related AEs (4.5%) occurred and few patients discontinued (4.8%) for any reason, including safety or tolerability. There was a numerically higher percentage of patients with opioid-related AEs in the placebo group compared with the active treatments. The etoricoxib 120 mg group also had the lowest proportion of patients with bleeding-related AEs among the treatment groups (Table 3).

**Table 3:** Summary of safety and tolerability

	**Placebo**	**Etoricoxib**	**Etoricoxibn**	**Ibuprofen**
	**N = 98**	**90 mg**	**120 mg**	**1800 mg**
	**n(%)**	**N = 222**	**N = 230**	**N = 223**
		**n(%)**	**n(%)**	**n(%)**
**One or more AEs**	68 (69.4)	141 (63.5)	144 (62.6)	134 (60.1)
Discontinued due to AEs	5 (5.1)	14 (6.3)	8 (3.5)	10 (4.5)
Drug-related AEs	3 (3.1)	12 (5.4)	11 (4.8)	9 (4.0)
Discontinued due to Drug-related AEs	1 (1.0)	2 (0.9)	1 (0.4)	3 (1.3)
Serious AEs	4 (4.1)	5 (2.3)	4 (1.7)	5 (2.2)
Discontinued due to Serious AEs	1 (1.0)	4 (1.8)	2 (0.9)	2 (0.9)
**Tier 1 AEs**				
CHF, pulmonary edema, or cardiac failure composite	0 (0.0)	1 (0.5)	0 (0.0)	0 (0.0)
Edema-related	4 (4.1)	2 (0.9)	4 (1.7)	4 (1.8)
Hypertension-related	3 (3.1)	8 (3.6)	3 (1.3)	7 (3.1)
Opioid-related	41 (41.8)	77 (34.7)	84 (36.5)	81 (36.3)
**Tier 2 AEs (AEs that occurred in ≥4 patients in any one treatment group)**				
Constipation	13 (13.3)	26 (11.7)	33 (14.3)	30 (13.5)
Dyspepsia	8 (8.2)	5 (2.3)	9 (3.9)	10 (4.5)
Nausea	31 (31.6)	50 (22.5)	56 (24.3)	53 (23.8)
Vomiting	13 (13.3)	27 (12.2)	30 (13.0)	33 (14.8)
Edema peripheral	4 (4.1)	2 (0.9)	4 (1.7)	4 (1.8)
Pyrexia	27 (27.6)	13 (5.9)	25 (10.9)	23 (10.3)
Anaemia (postoperative)	4 (4.1)	11 (5.0)	6 (2.6)	6 (2.7)
Dizziness	3 (3.1)	14 (6.3)	15 (6.5)	14 (6.3)
Headache	3 (3.1)	9 (4.1)	7 (3.0)	6 (2.7)
Insomnia	11 (11.2)	23 (10.4)	21 (9.1)	17 (7.6)
Oliguria	4 (4.1)	2 (0.9)	6 (2.6)	4 (1.8)
Hyperhidrosis	4 (4.1)	14 (6.3)	14 (6.1)	11 (4.9)
Pruritus	4 (4.1)	19 (8.6)	15 (6.5)	17 (7.6)
**AEs of Interest in Postoperative Patients**				
Bleeding-related AEs	7 (7.1)	16 (7.2)	11 (4.8)	14 (6.3)
Wound infection-related AEs	0 (0.0)	4 (1.8)	1 (0.4)	5 (2.2)
Wound-related AEs	2 (2.0)	1 (0.5)	2 (0.9)	2 (0.9)
**All Confirmed Thromboembolic Events**				
Myocardial Infarction	0 (0.0)	1 (0.5)	1 (0.4)	0 (0.0)
Transient Ischemic Attack	1 (1.0)	0 (0.0)	0 (0.0)	0 (0.0)
Pulmonary Embolism	0 (0.0)	1 (0.5)	1 (0.4)	0 (0.0)
Cardiac Arrest	1 (1.0)	0 (0.0)	0 (0.0)	0 (0.0)

Overall, 6 confirmed thromboembolic events occurred in 5 patients during the treatment period. There were 2 events (cardiac arrest and transient ischemic attack) in 1 patient in the placebo group. Two patients in the etoricoxib 90 mg group and 2 patients in the etoricoxib 120 mg group had cardiovascular events; in both groups, there was a patient with a pulmonary embolism and a patient with acute myocardial infarction. No confirmed thromboembolic events occurred in the ibuprofen group. Of note, both patients with myocardial infarction had pre-existing risk factors.

### Recovery index opioid side effect scale

**Figure 5:**
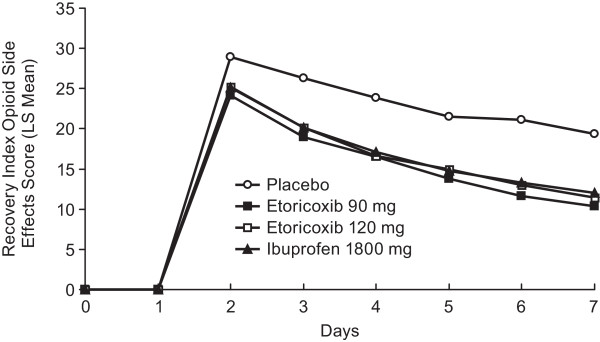
Recovery Index (Opioid Side Effects Scale) is shown over Days 2 to 7.

The LS mean score for the Recovery Index Opioid Side Effects Scale showed that both etoricoxib doses (120 mg, 90 mg) were consistently lower (patients reported less opioid-related side effects) than those on placebo, over the 7 days of the study; the LS mean scale scores were similar between the 2 etoricoxib dose groups and ibuprofen 1800 mg group (Figure 5).

## Discussion

Etoricoxib is a selective COX-2 inhibitor that has shown efficacy in the treatment of acute pain [11]. This randomized, double-blind trial evaluated the role of 2 doses of etoricoxib, 90 mg and 120 mg, in a multimodal analgesic regimen in patients who underwent unilateral knee replacement surgery. The goal of post-operative pain management is to improve patient satisfaction, reduce the length of the hospital stay, and overall facilitation of the rehabilitation process by providing pain relief and reducing opioid-related AEs [17].

In this trial, patients were provided with morphine through a PCA pump on the first day and opioids on subsequent days; etoricoxib 90 mg, etoricoxib 120 mg, ibuprofen 1800 mg, and placebo were provided to these patients in addition to the PCA pump and opioids. Although patients were able to titrate morphine use to the level of desired pain relief in all treatment groups, patients given etoricoxib demonstrated significantly lower pain intensity than patients given placebo and standard opioid-based analgesic treatment. Additionally, consumption of opioids was reduced in association with a reduction in patient-reported opioid negative consequences. The etoricoxib (120 mg, 90 mg) groups were non-inferior to ibuprofen 1800 mg/day for both the pain and morphine consumption endpoints (no formal testing was done to compare ibuprofen and placebo). The benefits of etoricoxib were observed consistently across multiple endpoints, including Pain Intensity at Rest, Pain Intensity with Knee Flexion, Total Daily Dose of Morphine consumption, and Opioid Side Effects Scale scores.

The results reported here are consistent with the level of pain relief reported in a recent meta-analysis of previous trials of selective COX-2 inhibitors with a total of 571 patients; the reduction in pain scores in the meta-analysis on a 0–10 visual analog scale ranged from -0.40 to -1.31 over the first 24 hours and 0.01 to -1.04 from 24 to 48 hours [18]. The differences from placebo in pain intensity for the present trial were -0.54 and -0.48 units for etoricoxib 90 and 120 mg, respectively, and -0.44 units with ibuprofen. The reduction of morphine in the meta-analysis ranged from -0.03 to -7.00 mg while the reduction in our trial was -4.53 and -4.24 mg for etoricoxib 90 and 120 mg, respectively, and -4.58 mg for ibuprofen [18]. Given the large size of the present trial, with 773 patients and its multi-site, multi-country conduct, the results would be expected to be more generalizable than smaller, regionally conducted trials evaluating analgesics in this clinical setting.

The results of the present trial confirm the results of an earlier, smaller trial examining the 120-mg dose of etoricoxib in the treatment of patients after knee or hip surgery; in that study, etoricoxib was superior to placebo and similar to the traditional NSAID, controlled-release naproxen sodium 1100 mg [11]. Etoricoxib 90 mg has been compared to etoricoxib 120 mg in two other prior post-operative pain settings. It was evaluated in the post-operative pain setting using third-molar extraction over a 3-day treatment period [19]. These two doses were also evaluated in patients undergoing total abdominal hysterectomy surgery over a treatment period of 5 days that included pre-operative dosing [20]. In those studies, etoricoxib 90 mg performed similarly to the 120-mg dose according to the primary endpoints used, although there was some evidence of separation of the two doses when evaluating efficacy upon movement endpoints in the abdominal hysterectomy trial [19,20].

As a methodologically interesting point, the benefits of etoricoxib became more evident beyond the first 2 days after surgery; the period of Days 4 to 7 demonstrated greater separation from placebo as compared with Days 1 to 3. This finding may have been due to the very high pain burden early postoperatively, and the larger requirement for morphine; the differences in reduction of pain intensity between etoricoxib and placebo increased as the use of opioids decreased. The improvements from baseline in Pain Intensity at Rest for both etoricoxib groups (120 mg, 90 mg) were numerically but not statistically greater than that of placebo on Day 1, when morphine use was the greatest. However, these differences increased over time for both etoricoxib groups, compared with placebo, over Days 2 to 7, as the pain burden and the subsequent morphine use declined. Morphine consumption was approximately 15% less for both etoricoxib groups (120 mg, 90 mg), compared with placebo, on Day 1, increasing to approximately 25% less on Day 2 and between 34% to 55% less on Days 3 to 7 in the etoricoxib groups. This pattern of greater separation from placebo over time was also observed in the Average Pain Intensity with Knee Flexion endpoint, an endpoint that also increased the pain stimulus as compared with assessing pain at rest. The etoricoxib 120-mg group showed greater reduction from baseline in average Pain Intensity with Knee Flexion compared with ibuprofen 1800 mg over the Day 4 to 7 interval.

This trial was conducted prior to the recommendations by the IMMPACT group for the evaluation of pain in trials [13]. We therefore performed *post hoc* responder analyses consistent with IMMPACT recommendations. These analyses demonstrated greater separation for the Average Pain Intensity with Knee Flexion endpoint among patients who achieved >70% pain improvement for etoricoxib 120 mg vs. etoricoxib 90 mg, ibuprofen, or placebo.

Subgroup analyses demonstrated that both subgroups of patients, those with severe pain and those patients with moderate pain, achieved a reduction in Pain Intensity at Rest as well as having a reduction in morphine consumption with etoricoxib as compared with placebo. Baseline pain was higher in patients with general anesthesia compared with spinal anesthesia; this observation may be important for future research with analgesic compounds as increased baseline pain may improve assay sensitivity and better identify any treatment differences. In this study, a higher proportion of patients had spinal anesthesia; with regard to assay sensitivity over Days 1 to 3, a larger difference from placebo was observed for general anesthesia for the endpoint of LS Geometric Mean Morphine Consumption.

The incidence of clinical AEs was generally similar among the placebo, etoricoxib (120 mg, 90 mg), and ibuprofen groups. There were no notable differences in serious AEs or discontinuations from AEs. Thromboembolic events are known perioperative complications of total knee replacement surgery, with preventative therapy recommended in most guidelines [21-23]. These events are also of interest for the evaluation of traditional NSAIDs and COX-2 selective inhibitors [24]. The incidence of myocardial infarction and pulmonary embolism in this study was similar to the incidence observed in a previous analysis of 10,244 patients who underwent primary total hip or knee arthroplasty [25]. Overall, the rate of cardiovascular AEs in this trial was low, was deemed unrelated to study medication by the investigators, and was consistent with the background rate of events postoperatively in this patient group. However, patients with certain cardiovascular comorbidities and risk factors were excluded from this trial.

Bleeding-related AEs and wound infection AEs were similar among both the placebo and the treatment groups. The evaluation of patient reporting of adverse experiences related to opioids, as measured by patient-reported Recovery Index Opioid Side Effects Scale demonstrated an important reduction in opioid-related AEs in the active treatment groups compared with the placebo group, documenting the intended benefit of multimodal pain care postoperatively. Because patients were permitted to use morphine/oxycodone *ad libitum* for pain control, achieving the simultaneous reduction in pain intensity at rest and a reduction in opioid use, with less patient-reported opioid negative experiences, reinforces the tenets of multimodal care and suggests that adding etoricoxib to standard opioid therapy has distinct, measureable advantages compared with an opioids-alone approach.

This was a multicenter study conducted in several countries; research has recently shown increasing the number of countries and sites increases the study variability, leading to different estimates of treatment effectiveness in osteoarthritis patients [26]. In the total knee replacement acute pain setting, an increase in study variability could occur due to different operation procedures, different anesthesia, and different postoperative care depending on standard procedures at individual sites and countries, which could confound results. An additional limitation for this study was that it was not powered to evaluate differences in bleeding risk between etoricoxib and ibuprofen given postoperatively, nor was it powered to detect differences in risk for cardiovascular events.

Nevertheless, the present trial is an important contribution to the literature for NSAIDs and selective COX-2 inhibitors in the post-operative setting due to several unique aspects. It is among the largest single trials conducted to evaluate acute pain in a postoperative setting over a period of 7 days with a 21-day follow-up period; notably, it is larger than a recent metaanalysis of all prior trials combined [18]. The 49-item Recovery Index and the 22-item Opioid Side Effects Scale are novel approaches for evaluating patient recovery. Finally, despite the aforementioned limitations due to the multicenter, multinational conduct, the completion of this trial demonstrates that large trials evaluating postoperative care across large geographic regions is possible and shows that the results are applicable across different nations and cultures despite widely different therapeutic regimens.

## Conclusions

In summary, in the treatment of pain following a unilateral total knee replacement, etoricoxib, at doses of 90 mg or 120 mg, administered postoperatively and then daily for the next 6 days on a background of opioid use resulted in greater pain control in this study as compared with placebo (representing opioid use alone), as evidenced by improvements in pain intensity at rest as well as leading to a reduction in opioid use, with its attendant reduction in patient-reported adverse experiences from opioids. The improvements in the etoricoxib 120- and 90-mg groups were non-inferior to the ibuprofen 1800 mg group. Importantly, it was found that both etoricoxib 90 mg and 120 mg doses had a similar tolerability and safety profile to placebo with better patient-reported tolerability on the opioid side effects scale.

## Abbreviations

AEs: Adverse events; ANOVA: Analysis of variance; APaT: All patients as treated; FAS: Full analysis set; IMMPACT: Initiative on methods, measurement, and pain assessment in clinical trials; LDA: Longitudinal data analysis; LS: Least squares; NRS: Numerical rating scale; NSAIDs: Non-steroidal anti-inflammatory drugs; PCA: Patient-controlled analgesia; RI: Recovery index.

## Competing interests

LC, SS, AM, DKC, SPC, DP are or were employees of Merck and may own stock or stock options in the company. NR has received lecture and consulting fees from Sintetica. EV has received lecture and consulting fees from Merck and has received payment for development of educational presentations from Merck. HM’s institution has received grants from Research Concepts GP LLC.

## Authors’ contributions

RN: Collected or assembled data; wrote sections of the initial draft and provided substantive suggestions for revision or critically reviewed subsequent iterations of the manuscript. EV: Collected or assembled data; wrote sections of the initial draft and provided substantive suggestions for revision or critically reviewed subsequent iterations of the manuscript. PMP: Conceived, designed or planned the study; performed or supervised analyses; interpreted the results; wrote sections of the initial draft and provided substantive suggestions for revision or critically reviewed subsequent iterations of the manuscript; obtained funding and provided administrative, technical, or logistic support. HSM: Collected or assembled data; provided substantive suggestions for revision or critically reviewed subsequent iterations of the manuscript; provided study materials or patients. LC: Performed or supervised analyses; provided substantive suggestions for revision or critically reviewed subsequent iterations of the manuscript; provided statistical expertise. SS: Conceived, designed or planned the study; provided substantive suggestions for revision or critically reviewed subsequent iterations of the manuscript; reviewed drafts of manuscript and provided feedback. AM: Interpreted the results; wrote sections of the initial draft; provided substantive suggestions for revision or critically reviewed subsequent iterations of the manuscript; provided administrative, logistical, and technical support. DKC: Conceived, designed or planned the study; provided substantive suggestions for revision or critically reviewed subsequent iterations of the manuscript. SPC: Conceived, designed or planned the study; provided substantive suggestions for revision or critically reviewed subsequent iterations of the manuscript. DAP: Performed or supervised analyses; interpreted the results; provided substantive suggestions for revision or critically reviewed subsequent iterations of the manuscript. All authors reviewed and approved the final version of the paper.

## Pre-publication history

The pre-publication history for this paper can be accessed here:

http://www.biomedcentral.com/1471-2474/14/300/prepub

## Supplementary Material

Additional file 1 List of Investigators and Independent Ethics Committees. Click here for file
