# Supplementary material for: Evaluation of etoricoxib in patients undergoing total knee replacement surgery in a double-blind, randomized controlled trial
Source: BMC Musculoskelet Disord. 2013 Oct 24;14:300. doi: 10.1186/1471-2474-14-300 (PMC3840772; doi:10.1186/1471-2474-14-300)
Supplement: Additional file 1 — List of Investigators and Independent Ethics Committees. [file 1471-2474-14-300-S1.pdf]

List of Investigators and Independent Ethics Committees and List of IEC Approvals Dates

Primary Investigator/Address	Protocol Study Number/Site Number	Sub-investigators	Name and Address Independent Ethics Committee (IEC)	Committee Chairperson/ Alternate	Number of Subjects Randomized	Date of IEC Approvals of Protocol/Amendments†
Kovacs, Tamas<br>Hospital of Zala County<br>Aneszteziologia<br>ZrinyiM.u.1<br>Zalaegerszeg 8900<br>Hungary	98000201	Laki, Zsolt	Egészségügyi Tudományos Tanács KFEB<br>Arany J. u. 6-8<br>Budapest 1051<br>Hungary	Furst, Zsuzsanna	14	098-01 02-Jun-09<br>098-02 11-Jun-10
Buvanendran, Asokumar<br>Rush University Medical Center<br>1485 Jelke 1725 W. Harrison St., Ste. 550<br>Chicago, IL 60612<br>USA	98000301	Della Vella Craig;<br>Rosenberg, Aaron	Rush University Medical Center<br>Rush Res and Clin Trials Administration 1725 W.Harrison St., Ste. 439<br>Chicago IL 60612<br>USA	Kravitz, Howard	6	098-01 23-Mar-09
Dowling, William<br>Morristown Memorial Hospital<br>100 Madison Avenue, Box 20<br>Morristown, NJ 07960<br>USA	98000401		Atlantic Health System IRB<br>Office of Grants and Research 475 South Street – Corporate<br>Morristown NJ 07962<br>USA	Dise, Craig	None	098-01 14-Jan-09
Jahr, Jonathan<br>UCLA Medical Center<br>D.G. School of Med., Dept. of Anesthesiology, Room 56-112<br>CHS 650 Charles Young Dr South, Box 957115<br>Los Angeles, CA 90095-7115<br>USA	98000501	Levin, Philip;<br>Sadoughi, Ali;<br>Zadeh, Bita H.	Western Institutional Review Board<br>3535 Seventh Avenue SW<br>Olympia WA 98502-5010<br>USA	Schultz, Theodore	6	098-01 06-May-09<br>098-02 11-Mar-10

Jones, Robert Kevin<br>Caring Clinical Research Corporation<br>24012 Calle de la Plaza, Suite 400<br>Laguna Hills, CA 92653<br>USA	98000601	Skinner, David	Memorial Health Services Research Council<br>2801 Atlantic Ave.<br>Long Beach CA 90806, USA	Patel, Ramesh	10	098-01 17-Nov-08<br>098-02 17-May-10
Singla, Neil K.<br>Huntington Memorial Hospital<br>100 West California Blvd.<br>Pasadena, CA 91105<br>USA	98000701	Farino, Ginamarie;<br>Savitala-Damerla;<br>Wright, Anne	Huntington Hospital IRB<br>Medical Staff Office 100 W. California Blvd<br>Pasadena CA 91105<br>USA.	Linsey, Michael	3	098-01 08-Dec-08<br>098-02 31-Mar-10
Najman, Stanislav<br>Klinika Dr. Pirka, s.r.o.<br>Na Celne 885<br>Mlada Boleslav, 293 01<br>Czech Republic	98000801		Multicentric EC Praha Motol<br>Vratislav Smelhaus, MD<br>V Uvalu 84<br>Prague 150 00,<br>Czech Republic	Smelhaus, Vratislav	106	098-01 11-Mar-09<br>098-02 07-Apr-10
Novacek, Jiri<br>Oblastni Nemocnice Mlada Boleslav<br>Klaudianova Nemocnice<br>V. Klementa 147<br>Mlada Boleslav, 293 50<br>Czech Republic	98000901		Multicentric EC Praha Motol<br>Vratislav Smelhaus, MD<br>V Uvalu 84<br>Prague 150 00,<br>Czech Republic	Smelhaus, Vratislav	10	098-01 25-Mar-09<br>098-02 28-Apr-10
Prochazka, Egon<br>FNHK, Ortopedicka klinika<br>Sokolska 581<br>Hradec Kralove, 500 05<br>Czech Republic	98001001		Multicentric EC Praha Motol<br>Vratislav Smelhaus, MD<br>V Uvalu 84<br>Prague 150 00,<br>Czech Republic	Smelhaus, Vratislav	24	098-01 05-Mar-09<br>098-02 01-Apr-10
Lo, Ngai Nung<br>Singapore General Hospital – Dept. of Orthopeaedics<br>Department of Orthopedic Surgery Block 6 Level 7<br>Outram Road	98001101	Chia, Shi Lu;<br>Chin, Pak Lin;<br>Yeo, Seng Jin	Singapore General Hospital<br>Institutional Review Board<br>Outram Road,<br>Singapore 169608	Eng, Aw Swee	None	098-01 15-Jan-09

Singapore 169608						
Altintas, Faik<br>Yeditepe University Faculty of Medicine<br>Department of Orthopedics and Traumatology<br>102/104 Kozyatagi 34752<br>Istanbul<br>Turkey	98001201	Ulucay, Cagatay<br>Yuksel, Korcan	Yeditepe Uni. Hospital, Devlet Yolu Ankara Cad. No:102/104 Kozyatagi,34752/ Istanbul	Gökçe , Özcan	7	098-01 03-Feb-09<br>098-02 17-Jan-11
Erdemli, Bulent<br>Ankara Universitesi<br>Faculty of Medicine,<br>Department of Orthopedics and Traumatology<br>Cebeci 06100<br>Ankara<br>Turkey	98001301	Kalem, Mahmut;<br>Karaduman, Mert	Ankara Uni. Fac. of Medicine, Sihhiye-06100,ANKARA	Ayhan , İsmail Hakkı	2	098-01 28-May-09<br>098-02 17-Jan-11
Sener, Ertugrul<br>Gazi University Faculty of Medicine, Department of Orthopedics and Traumatology<br>Besevler 06500<br>Ankara<br>Turkey	98001401	Dur, Hakan;<br>Kanatli, Ulunay;<br>Kulduk, Ahmet;<br>Yildirim Ahmet	Gazi Uni. Fac. of Medicine, Besevler – 06500, ANKARA	Buyan, Nejla	5	098-01 28-May-09<br>098-02 17-Jan-11
Alparslan, Mumtaz<br>Hacettepe University Faculty of Medicine, Department of Orthopedics and Traumatology<br>Sihhiye 06100, Ankara<br>Turkey	98001501	Atilla, Bulent;<br>Caglar, Omur	Yeditepe Uni. Hospital, Devlet Yolu Ankara Cad. No:102/104 Kozyatagi,34752/ Istanbul	Gökçe, Özcan	1	098-01 28-May-09<br>098-02 17-Jan-11
Tan, Ismet<br>Cukurova University Rectorate<br>Faculty of Medicine,<br>Department of Orthopedics and Traumatology<br>Merkez 01330, Adana,<br>Turkey	98001601	Ozkan, Cenk;<br>Tekin, Mustafa	Cukurova Uni. Fac. of Medicine, Balcali – 01330, ADANA	Uzel, Ilter	13	098-01 13-Feb-09<br>098-02 17-Jan-11

Van Zyl, Louis<br>Clinical Projects Research Center<br>42 Russell street<br>Worcester 6850<br>Western Cape<br>South Africa	98001701	Kiusmann, Karl; Johann Tredoux Enid N;<br>Van Dyk, Christo	Pharma Ethics,<br>123 Amcor Road<br>Lyttelton Manor,<br>Pretoria Gauteng 0157<br>South Africa	Duvenage, C S.J	7	098-01 29-Jan-09<br>098-02 03-Jun-10
Spargo, Catherine<br>Vincent Pallotti Hospital – Rheumatology Unit<br>Room 126, Alexandra Road<br>Pinelands 7405<br>Cape Town<br>South Africa	98001901	Bhorat, Rehana	Pharma Ethics,<br>123 Amcor Road<br>Lyttelton Manor,<br>Pretoria Gauteng 0157<br>South Africa	Duvenage, C S.J	None	098-01 29-Jan-09
McLennan-Smith, Rob<br>Westville Hospital, Suite 6A,<br>7 Spine Road, Westville<br>Durban, Kwazulu Natal 4001<br>South Africa	98002001	Macleod, Andrew	Pharma Ethics,<br>123 Amcor Road<br>Lyttelton Manor,<br>Pretoria Gauteng 0157<br>South Africa	Duvenage C S.J	54	098-01 29-Jan-09<br>098-02 03-Jun-10
Viscusi, Eugene<br>Thomas Jefferson University Hospital<br>Department of Anesthesiology<br>111 South 11th Street, Ste. G8490<br>Philadelphia, PA 19107-5092, USA	98002301		Thomas Jefferson University<br>1015 Chestnut St. Suite 1100<br>Philadelphia PA 19107, USA	Brock, David G.	None	098-01 28-May-09
Wang, Edward<br>Manila Doctors Hospital<br>708 Medical Arts Building<br>United Nations Avenue Manila<br>Philippines 1000	98002401	Ang, Reynaldo E.; Azores, Gregorio; Dela Cruz, Ruel; Macasaet, Evalyn T.	Ethics review Board-MDH<br>2/F Library, Manila Doctors Hospital<br>United Nations Avenue Manila<br>Philippines 1000	Collantes, Epifania V.	2	098-01 13-Feb-09<br>098-02 27-Apr-10
Chuang, (Brendan) Shih-Youeng<br>Tri-Service General Hospital<br>No.325, Sec.2,	98002501	Cherng Chen-Hwan	Tri-Service General Hospital<br>Room 5113,	Chih-Shung Wong	None	098-01 19-Jan-09

Chenggung Rd.,<br>Neihu District<br>Taipei 886<br>Taiwan			5F Medical Building, No. 325, Sec. 2, Chenggong Rd., Neihu District,<br>Taipei<br>Taiwan			
Huang, Teng-Le<br>China Medical University Hospital, Building, No. 2<br>Yude Rd.<br>Taichung 886<br>Taiwan	98002601	Fong, Yi-Chin;<br>Hsu, Horng-Chaung;<br>Wu (Rick) Sai-Chuen	Local Institutional review Board/IEC<br>9F First Medical building,<br>Taichung 40447<br>Taiwan	Wen-Liang Huang	15	098-01 22-Dec-08<br>098-02 02-Aug-10
Albrecht, Stephan<br>Ev. Waldkrankenhaus Spandau<br>Abteilung fuer Orthopaedie<br>Stadtrandstr.555-561<br>Berlin 13589<br>Germany	98002701		EK der Bayerischen Landesaerztekammer<br>Mühlbaurstraße 16<br>München 81677<br>Germany	Hasford, Joerg	None	098-01 18-Jun-09<br>098-02 23-Apr-10
Hellinger, Stefan<br>Praxis Dr. med. Stefan Hellinger<br>Facharzt fuer Orthopädie und orthopädische Chirurgie<br>Windenmacherstr. 2 München 80333<br>Germany	98002801		EK der Bayerischen Landesaerztekammer<br>Mühlbaurstraße 16<br>München 81677<br>Germany	Hasford, Joerg	4	098-01 18-Jun-09<br>098-02 23-Apr-10
Kelk, Margus<br>West-Tallinn Central Hospital<br>Orthopaedic Department<br>Sole 16<br>Tallinn 1061<br>Estonia	98003001	Maripuu, Irja;<br>Uhtegi, Krista	Tallinn Medical Research Ethics Committee<br>Hiiu 42<br>Tallinn Eesti 11619<br>Estonia	Põlluste, Jaak	20	098-01 12-Mar-09<br>098-02 15-Apr-10
Martson, Aare,<br>Univeristy of Tartu<br>Hospital Traumatology and Orthopaedics Clinic<br>8L. Puusepa Street<br>Tartu 51014	98003101	Kolk, Helgi;<br>Parv, Mart;<br>Paul, Sigrid;<br>Puuorg, Egon;<br>Rull, Alo	Tallinn Medical Research Ethics Committee<br>Hiiu 42<br>Tallinn Eesti 11619<br>Estonia	Põlluste, Jaak	45	098-01 12-Mar-09<br>098-02 15-Apr-10

Estonia						
Oeding, Oscar<br>Hospital Clinica Biblica<br>Calle 2ePrimera entre Ave 14-16<br>San Jose<br>Costa Rica	98003201	Alvarado, Randall;<br>Aviles, Ronier	Comité Etico Científico Universidad de Ciencias Médicas<br>De la Pops de Sabana 400 m Oeste Contiguo a las of de AMNET<br>San José San José N/A<br>Costa Rica	De Céspedes Montealegre Carlos	1	098-01 31-Mar-09<br>098-02 16-Jul-10
Filip, Grevbo<br>Sykehuset Gjøvik akuttmed<br>Kyrre Greppsgate 11<br>Gjovik, 2819<br>Norway	98003501	Kjernlie, Dag Frode	Rek Sor-Ost<br>Postboks 1130 Blindern<br>Oslo, Norway 0318	Heiberg Arvid	2	098-01 23-Mar-09
Ivaskevicius, Juozas<br>Vilniaus Greitosios Pagalbos Ligonine<br>Siltnamiu Str.29<br>Vilnius LT-04130, Lithuania<br>Lithuania	98003601	Kocius, Manvilius	Lietuvos Bioetikos Komitetas<br>Didzioji g. 22<br>Vilnius LT-01128, Lithuania	Gefenas, Eugenijus	36	098-01 10-Feb-09<br>098-02 04-May-10
Karbonskiene, Aurika<br>Lietuvos Sveikatos Mokslu Universiteto<br>Ligonine Kauno Klinikos<br>Eiveniu 2<br>Kaunas 50010<br>Lithuania	98003701	Gelmanas, Arunas	Lietuvos Bioetikos Komitetas<br>Didzioji g. 22<br>Vilnius LT-01128, Lithuania	Gefenas, Eugenijus	23	098-01 10-Feb-09<br>098-02 04-May-10
Minkowitz, Harold S.<br>Memorial Hermann Memorial City Hospital<br>Anesthesia Department<br>Room F<br>921 Gessner Road<br>Houston, TX 77054<br>USA	98003801		Western Institutional review Board<br>3535 Seventh Avenue SW<br>Olympia WA 98502-5010, USA	Schultz, Theodore	67	098-01 07-May-09<br>098-02 11-Mar-10
Singla, Neil K.<br>HuntingtonMemorial Hospital	98003901	Farino, Ginamarie;	Western Institutional review Board	Schultz, Theodore	None	098-01 29-Apr-09

100 West California Blvd.<br>Pasadena 91105, CA<br>USA		Savitala-Damerla	3535 Seventh Avenue SW<br>Olympia WA 98502-5010, USA			
Szerb, Imre<br>Fövárosi Önkormanyzat Uzsoki Utcai Korzhaz Ortopéd-traumatologia Urzoki u. 29-45<br>Budapest 1145<br>Hungary	98004001	Cseri, Zoltan	Egészségügyi Tudományos Tanács KFEB<br>Arany J. u. 6-8<br>Budapest 1051, Hungary	Furst, Zsuzsanna MD	10	098-01 02-Jun-09<br>098-02 11-Jun-10
Azores, Gregorio<br>Philippine General Hospital<br>Taft Avenue<br>Manila 1000<br>Philippines	98004101	Barrion, Aimee Flor; Ang, Reynaldo E.; Bernardo, Peter; Cruz, Patricia Lorna; Montalban Jr., Antonio	RIDO_Ethics Review Board<br>Research Implementation & Dev. Ofc<br>#547 Pedro Gill St.<br>Ermita, Manila 1000<br>Philippines	Santos, Evangeline Olivar;<br>Vios, Salome M.	3	098-01 19-May-09<br>098-02 21-Apr-10
Andreas M, Halder<br>Sana Kliniken Sommerfeld<br>Hellmuth-Ulrici-Kliniken<br>Sommerfeld Klinik fur Endoprothetik<br>Waldhausstrasse<br>Kremmen OT Sommerfeld, 16766<br>Germany	98004201		EK der Bayerischen Landesaerztekammer<br>Mühlbaurstraße 16<br>München 81677<br>Germany	Hasford, Joerg	10	098-01 18-Jun-09<br>098-02 23-Apr-10
Kostka, Andreas/ Muschik, Michael<br>Park-Klinik Weissensee<br>Klinik fur Orthopadie<br>Schonsrabe 80<br>Berlin 13086<br>Germany	98004401		EK der Bayerischen Landesaerztekammer<br>Mühlbaurstraße 16<br>München 81677<br>Germany	Hasford, Joerg	9	098-01 18-Jun-09<br>098-02 23-Apr-10
Hans-Georg, Fieseler<br>ZOC- Zentrum Orthopaedische Chirurgie<br>Burckhardtstrasse 60	98004501		EK der Bayerischen Landesaerztekammer<br>Mühlbaurstraße 16<br>München 81677	Hasford Joerg	18	098-01 18-Jun-09<br>098-02 23-Apr-10

Hans Munden 34346<br>Germany			Germany			
Chelly, Jacques<br>UPMC Shadyside<br>Aiken Medical building<br>532 South Aiken Avenue, Suite 407<br>Pittsburgh, PA 15232<br>USA	98004601	Ben-Ari, Alon; Ben-David, Bruce; Biagini, Todd; Boretsky, Karen; Cain, Brent; Jakymec, Andres; Josh, Rama M.; Kandel, Arie; Khanzada, Mohammed; Khetarpal, Sharad K.; Marr, Larry; Merman, Rita B.; Rest, Carl; Sabo, Daniel; Uskova, Anna a.; Yennam, Sudharakar R.	Western Institutional review Board<br>3535 Seventh Avenue SW<br>Olympia WA 98502-5010, USA	Schultz, Theodore	1	098-01 24-Jun-09<br>098-02 11-Mar-10
Miller, Howard<br>Research Concepts, Ltd.<br>7800 Fannin, Suite 205<br>Houston, TX 77054<br>USA	98004801	Rechter, Alan	Western Institutional review Board<br>3535 Seventh Avenue SW<br>Olympia WA 98502-5010, USA	Schultz, Theodoro	None	098-01 11-Jun-09<br>098-02 11-Mar-10
Shannon, Kelly<br>UPMC St. Margaret<br>815 Freeport Rd., Office 1512<br>Pittsburgh, PA 15215<br>USA	98004901	Richrds, Charles; Roskoph, Jay; Taormina, Darrin	Western Institutional review Board<br>3535 Seventh Avenue SW<br>Olympia WA 98502-5010, USA	Schultz, Theodoro	None	098-01 10-Jun-09<br>098-02 11-Mar-10
Kurth, Andreas<br>Klinikum der Johann Wofgang	98005001		EK der Bayerischen Landesaerztekammer	Hasford, Joerg	1	098-01 18-Jun-09<br>098-02 23-Apr-10

Gothe-Universitat<br>Orthopaedische Klinik und Poliklinik<br>Langenbeckstrasse 1<br>Mainz 55131<br>Germany			Mühlbaurstraße 16<br>München 81677<br>Germany			
Lierz, Peter<br>Marienkrankenhaus Sost<br>Fachbereich fur Anaesthesiologie und Intensivmedizin<br>Widumgabe 5<br>Soest 59494<br>Germany	98005101	Losch, Holger; Steinbrich, Roman; Strunk, Hedwig	EK der Bayerischen Landesaerztekammer<br>Mühlbaurstraße 16<br>München 81677<br>Germany	Hasford, Joerg	None	098-01 18-Jun-09<br>098-02 23-Apr-10
Lohmann, Klinikum Bad Bramstedt<br>Klinik fur Orthopaedie Oskar-Alexander-Strasse 26<br>Bad Bramstedt 24576<br>Germany	98005201	Wenk, Wanja	EK der Bayerischen Landesaerztekammer<br>Mühlbaurstraße 16<br>München 81677<br>Germany	Hasford, Joerg	15	098-01 18-Jun-09<br>098-02 23-Apr-10
Daniel, Dobbert<br>Staedtisches Klinikum Dessau<br>Orthopaedie und Unfallchirurgie<br>Auenweg 38<br>Dessau 06847<br>Germany	98005401		EK der Bayerischen Landesaerztekammer<br>Mühlbaurstraße 16<br>München 81677<br>Germany	Hasford, Joerg	None	098-01 18-Jun-09<br>098-02 23-Apr-10
Singer, Robert J.<br>Blair Orthopedic Associates<br>3000 Fairway Drive<br>Altoona PA 16602<br>USA	98005501	Port, Joshua; Rowe, Angela	Blair Orthopedics<br>Altoona Regional Health System Inst. Review Committee-Med Staff office 620 Howard Ave.<br>Altoona PA 16602<br>USA	Drass, Michael J.	14	098-01 29-Jul-09<br>098-02 28-Apr-10
Kramberger, Slavko/Bordnik, Tomaz<br>Univerzitetni Klinicni center Maribor	98005601	Krajnc, Zmago; Molicnik, Andrej	Komisija za medicinsko etiko<br>Institut za nevrofiziologijo, Klinicni	Trontelj, Joze	38	098-01 22-Sep-09<br>098-02 17-Jun-10

Oddelek za ortopedijo<br>Ljublijanska ulica 5<br>Maribor 2000<br>Slovenia			center Ljubljana, Zaloska 7<br>Ljubljana 1525, Slovenia			
Bin, Seong II<br>Asan Medical Center<br>Department of Orthopedics<br>388-1 Poongnap-2dong<br>Songpa-gu<br>Seoul 138-736<br>Korea, Republic of	98005701	Lee, Bum Sik;<br>Lee, Sandg Jin;<br>Yang, Hong Seuk	Asan Medical Center<br>1F, IRB,Clinical Research Center<br>Seoul 138-736<br>Korea, Republic of	Lee, Sang Goo	15	098-01 14-Sep-09<br>098-02 03-May-10
Moon, Young-Wan<br>Samsung Medical Center<br>50, Ilwon-Dong,<br>Kangnam-Ku 135-710<br>Seoul,<br>Korea, Republic of	98005901	Do Kwan Hong; Heo Jae Won;<br>Kim, Jae Gyoon;<br>Kim, Jung Hwan;<br>Lee, Sung Sahn;<br>Shim Jae Woo;<br>Yim Hyun Seok	Asan Medical Center<br>1F, IRB,Clinical Research Center<br>Seoul 138-736<br>Korea, Republic of	Lee, Sang Goo	13	098-01 08-Oct-09<br>098-02 06-May-10
Ustaoglu, Gur<br>Izmir Ataturk Education and Research Hospital<br>Orthopedics and Traumatology Clinic<br>Basin Sitesi Yesilyurt<br>Izmir 35360<br>Turkey	98006001	Gumus, Bilal;<br>Yilmaz, Ferit	Istanbul Uni. Istanbul Fac. of Medicine Capa-Fatih/Istanbul, 34380	Uresin, A. Yağız	22	098-01 03-Feb-09<br>098-02 17-Jan-11
Lenart, Endre<br>Bacs-Kiskun Megyei Onkormanizat Korhaza<br>Nyiri ut 38<br>Kecskemet 6000<br>Hungary	98006201	Toth, Gabor	Egészségügyi Tudományos Tanács KFEB<br>Arany J. u. 6-8<br>Budapest 1051, Hungary	Furst, Zsuzsanna MD	10	098-01 02-Jun-09<br>098-02 11-Jun-10

Thornton, Iain<br>Sandton Medi-Clinic<br>Origin Clinical Research<br>Suite 207A South Block<br>Peter Place and Main Avenue<br>Johannesburg, Gauteng 2060<br>South Africa	98006301	Adler, David;<br>Botha, Julia;<br>Khonje Robert	Pharma Ethics,<br>123 Amcor Road<br>Lyttelton Manor,<br>Pretoria Gauteng 0157<br>South Africa	Duvenage, C S.J	None	098-01 20-Oct-09<br>098-02 03-Jun-10
Schmidt, David<br>Sports Medicine Associates of San Antonio<br>21 Spurs Lane, Suite 300<br>San Antonio, TX 78240<br>USA	98006601	Curtis, Ralph (Bud) J.;<br>Lozano, Michael E.;<br>Palomera, Timothy S.;<br>Peterson, Johnathan<br>Saenz, Paul;<br>Steffen, Richard T.	Western Institutional review Board<br>3535 Seventh Avenue SW<br>Olympia WA 98502-5010, USA	Schultz, Theodore	28	098-01 09-Feb-10<br>098-02 11-Mar-10
San Pedro, Paul Cesar N.<br>Philippine Orthopedic Center<br>Rm 8 Adult Orthopedic Clinic<br>Maria Clara St., co Benawe St.<br>Quezon City 1100<br>Philippines	98006701	Macaranas, Eugene;<br>Mutia Karlou Dinglasa<br>Ramirez, Mark;<br>Villanueva, Brenda	POC-Ethics Review Boar<br>Ethics Review Board,<br>Researh Ofc<br>Mezzanine Floor, Doctors' Ofc<br>Ma. Clara Cor. Benawe St.<br>Quezon City 1100, Philippines	Canete, Arturo C.	10	098-01 05-Jan-09<br>098-02 20-Jul-10
Yablanski, Vasil<br>MHAT - Toduka Hospital Sofia AD<br>Dept of Orthopedics and Traumatology<br>51 B Nikola Vaptzarov blvd.<br>Sofia 1407<br>Bulgaria	98006801	Pashov, Stanislav	Central Institutional Board/IEC<br>6 Damian Gruev Str.<br>Sofia 1303<br>Bulgaria	Alexander, Yankev	6	098-02 09-Sep-10
Yankov, Encho<br>Umhat Tsaritsa Yoanna	98006901	Fudulski, Alexander;	Central Institutional Board/IEC	Alexander, Yankev	1	098-02 09-Sep-10

Clinic of Orthopaedics and Traumatology<br>8, Byalo more Str.<br>Sofia 1527<br>Bulgaria		Kinov, Plamen	6 Damian Gruev Str.<br>Sofia 1303<br>Bulgaria			
Kosev, Pencho<br>MHAT Russe<br>Clinic of Orthopaedics<br>2, Nezavisimost Str.<br>Russe 7002<br>Bulgaria	98007001	Petrov, Vladin;<br>Sokolov, Tzvetan	Central Institutional Board/IEC<br>6 Damian Gruev Str.<br>Sofia 1303<br>Bulgaria	Alexander, Yankev	7	098-02 09-Sep-10
Manojlovic, Radovan<br>Clinical Center of Serbia<br>Institute of Orthopedic Surgery and Traumatology Visegradska 26<br>Belgrade 11000<br>Serbia	98007201	Bumbasirevic, Marko;<br>Kadija, Marko;<br>Lesic, Aleksandar;<br>Mihajiovic, Jovan;<br>Tabakovic, Dejan;<br>Tulic, Goran	Clinical center of Serbia-Ethics Committee<br>Pasterova 2<br>Belgrade Serbia 11000<br>Serbia	Maksimovic, Zivan	11	098-02 18-Mar-10
Cobeljic, Goran<br>Institut za Orthopedske Bolesti Banjica<br>Mihaila Avramovica 28<br>Belgrande 11000<br>Serbia	98007301	Djuric, Milena Z<br>Vukotic, Milovan	Institute Banjica Ethics Committee<br>Mihaila Avramovica 28<br>Belgrade Serbia 11000<br>Serbia	Nikolic, Olga	12	098-02 30-Mar-10
Rossmanov, Ventzi<br>SHATOTS Clinic of Orthopeadics and Traumatology<br>24 Trite bora Str.<br>Pleven 5800<br>Bulgaria	98007401	Nenov, Plamen	Central Institutional Board/IEC<br>6 Damian Gruev Str.<br>Sofia 1303<br>Bulgaria	Alexander, Yankev	1	098-02 09-Sep-10
Stavrev, Vladimir<br>Sveti Georgi Multiprofile Hospital for Active Treatment	98007501	Karamitev, Stanislav	Central Institutional Board/IEC<br>6 Damian Gruev Str.	Alexander, Yankev	5	098-02 09-Sep-10

Clinic of Orthopeadics and Traumatology<br>81 Peshtersko Shose Boulevard<br>Plovdiv 4000<br>Bulgaria			Sofia 1303<br>Bulgaria			
van der Plank, Robert<br>Olivedale Clinic<br>President Fouche Drive<br>Johannesburg, Gauteng 2188<br>South Africa	98007601	Blou, Michelle; Thornton, Iain	Pharma Ethics,<br>123 Amcor Road<br>Lyttelton Manor,<br>Pretoria Gauteng 0157<br>South Africa	Duvenage, C S.J	4	098-01 29-Apr-09<br>098-02 03-Jun-10
Fokter, Samo K.<br>General Hospital Celje<br>Oddelek za Ortopedijo in Sportne Poskodbe<br>Oblakova ulica 5<br>Celje 3000<br>Slovenia	98007701	Bukovnik, Igor; Strahovnik , Andrej	Komisija za medicinsko etiko<br>Institut za nevrofiziologijo, Klinicni center Ljubljana, Zaloska 7<br>Ljubljana 1525, Slovenia	Trontelj, Joze	17	098-01 22-Sep-09<br>098-02 17-Jun-10
Lohmann, Christoph<br>Otto-von-Guericke-Universitat Magdeburg<br>Medizinishe Fakultat<br>Orthopadische Universitasklinik<br>Haus 8<br>Leipziger Strasse 44<br>Magdeburg, 39120<br>Germany	98007801		EK der Bayerischen Landesaerztekammer<br>Mühlbaurstraße 16<br>München 81677<br>Germany	Hasford, Joerg	12	098-01 18-Jun-09<br>098-02 23-Apr-10

† Amendment 3 was administrative in nature and was approved on 25-Jan-11, after last patient last visit was completed. A summary of changes was submitted to IRBs but no formal approval was obtained.
